# Edible Mushrooms as Emerging Prebiotic Sources: Gut Microbiota Modulation and SCFA-Mediated Health Effects

**DOI:** 10.3390/foods15091539

**Published:** 2026-04-29

**Authors:** Laura Beatrice Mattioli, Luca Camarda, Martina Aicardi, Enrica Pasquali, Ivan Corazza, Roberta Budriesi

**Affiliations:** 1 Food Chemistry and Nutraceutical Lab, Department of Pharmacy and Biotechnology (FaBiT), Alma Mater Studiorum-University of Bologna, Via Belmeloro 6, 40126 Bologna, Italy; laurabeatrice.mattioli@unibo.it (L.B.M.); martina.aicardi3@unibo.it (M.A.); enrica.pasquali@studio.unibo.it (E.P.); roberta.budriesi@unibo.it (R.B.); 2 Health Sciences and Technologies-Interdepartmental Center for Industrial Research (CIRI-SDV), Alma Mater Studiorum-University of Bologna, Via Massarenti 9, 40126 Bologna, Italy; ivan.corazza@unibo.it; 3 Department of Medical and Surgical Sciences (DIMEC), Alma Mater Studiorum-University of Bologna, 40138 Bologna, Italy

**Keywords:** medicinal mushrooms, edible mushrooms, prebiotics, gut microbiota, short-chain fatty acids, β-glucans, bioactive compounds, gut–brain axis, functional foods, microbiota modulation

## Abstract

**Background**: Edible and medicinal mushrooms have attracted growing attention as functional foods due to their rich content of bioactive compounds and their potential to modulate host physiology through microbiota-mediated mechanisms. **Methods**: This narrative review was conducted through a comprehensive literature search across major scientific databases, including PubMed, Scopus, ScienceDirect, Web of Science, and Google Scholar, selecting studies focused on mushroom-derived compounds, gut microbiota, short-chain fatty acids (SCFAs), and the gut–brain axis (GBA). **Results**: Current evidence indicates that mushroom-derived polysaccharides, particularly β-glucans, along with polyphenols, trehalose, and chitin, resist digestion and are fermented by intestinal microorganisms, promoting SCFA production. These metabolites contribute to intestinal barrier integrity, immune regulation, and metabolic homeostasis and may also influence neuroinflammation and neurotransmitter pathways via the GBA. However, significant variability in mushroom preparations and the limited availability of well-designed human clinical trials remain important limitations. **Conclusions**: Edible and medicinal mushrooms represent a promising source of novel prebiotic compounds with potential systemic health benefits, although further standardized studies and robust clinical trials are needed to confirm their efficacy and mechanisms of action.

## 1. Introduction

When referring to medicinal mushrooms, we indicate specific fungal species that have been selected and used for centuries, commonly known as macrofungi [1]. From a taxonomic perspective, fungi belong to a kingdom distinct from both animals and plants within the supergroup Opisthokonta [2], a classification that reflects their unique biological and functional characteristics [3]. From an evolutionary standpoint, fungi are more closely related to animals than to plants [2]. This proximity is considered relevant to their biological activity, as fungi interact with microbial ecosystems and pathogens comparable to those affecting humans [1,4]. A well-known example is *Penicillium notatum*, from which Alexander Fleming isolated penicillin, the antibiotic that revolutionized medicine and marked the beginning of the antibiotic era [5].

The use of fungal-derived natural products has been documented since ancient times, as suggested by findings in the medical kit of the Similaun Man (Ötzi), which have been the subject of several scientific investigations [6,7].

In recent decades, edible and medicinal mushrooms have gained increasing attention as functional foods and nutraceuticals due to their rich content of biologically active compounds, including polysaccharides, polyphenols, terpenoids, and other secondary metabolites [8,9]. These molecules have been associated with several beneficial effects, including immunomodulatory, antioxidant, and metabolic regulatory activities [10,11]. Traditionally, the therapeutic use of mushrooms has been widely described in Traditional Chinese Medicine (TCM), where they are considered tonics that restore physiological balance and support systemic health [12,13,14]. In modern biomedical research, these complex biological effects are increasingly interpreted through the framework of network pharmacology, which investigates how multiple bioactive compounds can simultaneously interact with different biological targets and pathways [14,15].

**Figure 1:**
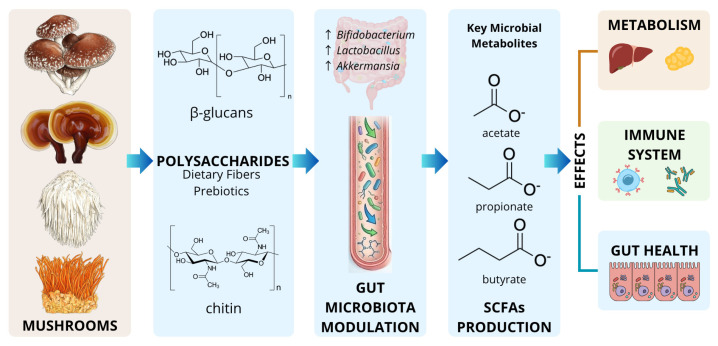
Schematic representation of the proposed interactions between mushroom-derived bioactive compounds, gut microbiota, and their potential physiological effects. Mushroom polysaccharides (e.g., β-glucans, chitin-derived oligosaccharides) may serve as fermentable substrates for gut microorganisms, promoting the growth of beneficial taxa such as *Bifidobacterium*, *Lactobacillus*, and *Akkermansia*. Microbial fermentation leads to the production of SCFAs, including acetate, propionate, and butyrate, which contribute to the maintenance of intestinal barrier integrity, modulation of immune responses and metabolic regulation.

Among the various mechanisms proposed to explain their health-promoting properties, the modulation of the gut microbiota has emerged as a key factor. Mushrooms contain several compounds capable of acting as prebiotic substrates, including high-molecular-weight β-glucans, polyphenols, trehalose, and chitin [16,17]. These components can promote microbial diversity and stimulate the production of short-chain fatty acids (SCFAs), metabolites that play important roles in intestinal homeostasis, metabolic regulation, and immune function (Figure 1). Through these mechanisms, mushrooms may help maintain a balanced microbial ecosystem and regulate low-grade systemic inflammation [18].

The gut microbiota is also a central component of the gut–brain axis (GBA), a bidirectional communication system that links the gastrointestinal tract to the central nervous system [19]. By influencing microbial composition and metabolite production, mushrooms may indirectly affect several physiological processes, including immune responses, metabolic balance, and the neurochemical signaling involved in mood and cognitive functions [20].

Although numerous edible and medicinal mushroom species exist, current scientific evidence regarding gut microbiota modulation and SCFA production is largely concentrated on a small number of well-studied species [21]. In particular, much of the available research focuses on mushrooms traditionally used in TCM, such as *Ganoderma lucidum*, *Hericium erinaceus*, *Cordyceps* spp., and *Lentinula edodes* [22], which are also widely consumed and commercially available worldwide as dietary supplements and functional foods [23].

Therefore, this review focuses on these extensively studied species, as they are the best-documented models for investigating the microbiota-mediated effects of mushroom-derived compounds.

In this context, the aim of the present review is to provide an overview of the role of edible and medicinal mushrooms as emerging prebiotic foods, with particular attention to the activity of β-glucans and other bioactive compounds in modulating the gut microbiota and promoting the production of SCFAs, as well as their potential implications for intestinal and systemic health [3,24].

## 2. Materials and Methods

This narrative review was conducted through three main phases: literature search, screening of abstracts and full texts, and critical analysis of the selected studies.

The literature search was performed using major scientific databases, including PubMed, Scopus, ScienceDirect, Web of Science, and Google Scholar, in order to identify relevant studies addressing the relationship between medicinal mushrooms, gut microbiota, and the GBA.

The search strategy combined primary keywords such as “medicinal mushrooms”, “edible mushrooms”, “prebiotics”, “gut microbiota”, “short-chain fatty acids”, and “gut–brain axis” with additional terms including “β-glucans”, “polyphenols”, “trehalose”, “chitin”, “metabolites”, “neuroinflammation”, “*Ganoderma lucidum*”, “*Hericium erinaceus*”, “Cordyceps”, and “*Lentinula edodes*”. Keywords were used individually and in combination to maximize the identification of relevant literature.

Following the initial search, duplicate records were removed, and titles and abstracts were screened to evaluate their relevance to the scope of the review. When necessary, full texts were assessed for eligibility. Studies were included if they reported experimental evidence (in vitro or in vivo), clinical studies, or comprehensive reviews addressing the effects of mushroom-derived compounds on gut microbiota composition, microbial metabolites, SCFA production, and host physiological responses related to the GBA.

Preference was given to peer-reviewed articles published in English within the past 10 years, in order to capture the most recent advances in the field. Nevertheless, earlier seminal publications were also considered when necessary to provide historical context or mechanistic insights.

**Figure 2:**
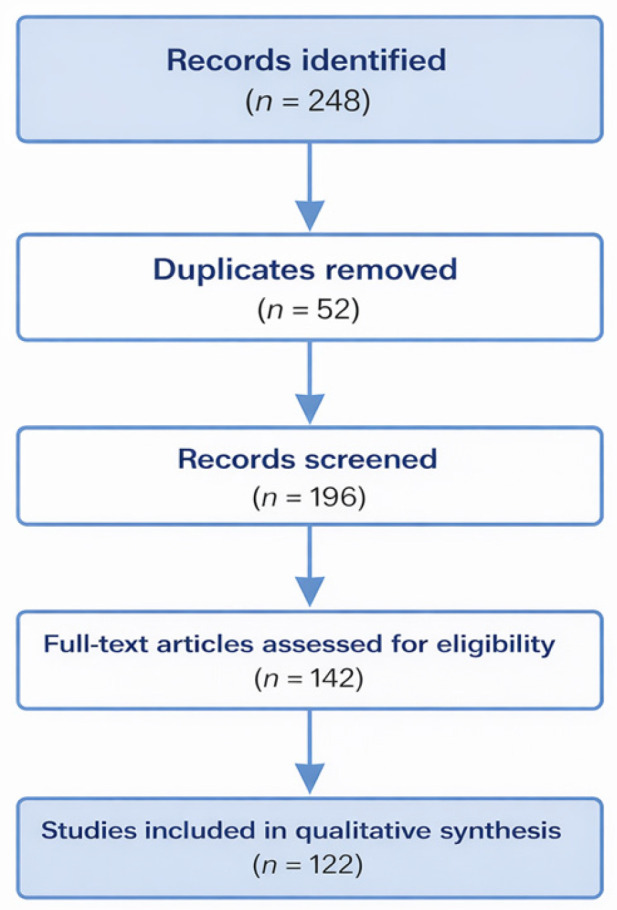
PRISMA-inspired flow diagram of the literature selection process used to identify the studies included in this narrative review.

Although this work was designed as a narrative review, a semi-structured literature selection approach was adopted to improve transparency and reproducibility. The study identification and selection process followed a PRISMA-inspired workflow, and a flow diagram summarizing the literature selection procedure is included in the revised manuscript (Figure 2).

Because of the narrative nature of this review, formal quality scoring systems typically used in systematic reviews were not applied. However, particular attention was given to methodological robustness, relevance to the gut microbiota–GBA, and the consistency of the reported biological effects when selecting and discussing the included studies.

## 3. Mushroom Preparations and Quality Considerations

From an anatomical perspective, *macrofungi* are composed of two main structural components: the mycelium and the fruiting body [25,26]. The mycelium is the vegetative portion of the fungus and consists of a complex network of microscopic, filamentous structures called hyphae [27,28]. These hyphae penetrate the growth substrate—such as soil, wood, or other organic matter—where they absorb nutrients required for fungal growth and metabolism. By secreting extracellular enzymes, the mycelium degrades complex organic substrates and absorbs the resulting nutrients, serving as the primary metabolic interface between the fungus and its environment [29].

In contrast, the fruiting body is the visible reproductive structure that emerges above the substrate when environmental conditions are favorable. Its primary biological function is the production and dispersal of spores, allowing the propagation of the fungal species [26,29].

At the cellular level, both the mycelium and the fruiting body are characterized by a robust cell wall mainly composed of structural polysaccharides such as chitin and β-glucans [30,31]. These polymers provide mechanical stability and protection while also contributing to many of the biological activities attributed to edible and medicinal mushrooms. In particular, β-glucans are among the most studied classes of mushroom-derived polysaccharides due to their immunomodulatory and microbiota-modulating properties. Understanding the structural organization of fungal biomass is therefore essential for interpreting the biochemical composition and biological activity of mushroom-based preparations [32,33].

### 3.1. Whole Mushroom, Extracts, and Combined Formulations

Due to the biochemical differences between fungal structures and the various processing methods used in nutraceutical preparations, the form of administration plays a crucial role in determining the biological effects of medicinal mushrooms.

Consuming the whole mushroom, including both the mycelium and the fruiting body, is the most complete way to obtain the natural fungal phytocomplex. This preparation allows the simultaneous intake of polysaccharides, lipid fractions, and insoluble fibers [10]. Because mycelium and fruiting body exhibit different metabolic profiles and concentrations of bioactive compounds, using the entire fungal biomass ensures the presence of complementary biochemical components and helps preserve the natural synergy among molecules [32].

The use of mushroom extracts is typically preferred when the objective is to obtain a higher concentration of specific classes of bioactive molecules. Because the extraction solvent selectively isolates compounds based on their solubility, aqueous extracts are generally enriched in polysaccharides, which are often associated with immunomodulatory effects, whereas ethanolic or lipophilic extracts enable the isolation of low-molecular-weight compounds such as terpenoids, which may act on more specific biological targets [8,34].

To combine the benefits of both approaches, combined formulations have been developed. These products link the entire mushroom biomass with a variable amount of concentrated extracts, maintaining the enzymatic integrity and synergistic interactions of the fungal phytocomplex while also offering higher concentrations of specific active compounds [35,36]. Additionally, the insoluble fibers present in the whole mushroom biomass may protect bioactive compounds from gastric and enzymatic degradation [13,37]. Their interaction with the gut microbiota may also contribute to a healthier intestinal environment, potentially improving the bioavailability of bioactive molecules in associated extracts and exerting direct health-promoting effects [22].

From a biochemical and functional perspective, mycelium and fruiting body are not equivalent. The fruiting body is generally richer in structural polysaccharides and antioxidant molecules, including polyphenols and other secondary metabolites [17]. In contrast, the developing mycelial network may produce specific metabolites involved in substrate colonization and environmental adaptation, some of which may be less abundant or absent in the mature fruiting structure [38]. Due to this biochemical variability, the biological activity and potential therapeutic effects of mushroom preparations may vary depending on the fungal component used and the processing methods applied [39].

In addition, commercial mushroom supplements may contain preparations derived from mycelium cultivated on grain substrates, which may include residual growth medium. This factor may contribute to variability in the biochemical composition and concentration of bioactive compounds in the final product [40].

### 3.2. Quality and Safety of Mushroom Preparations

The growing popularity of medicinal mushrooms has increased the need for careful evaluation of the quality and safety of commercial preparations [41]. The safety of mushroom-based supplements depends not only on processing methods but also on the purity and traceability of the raw material.

Mushrooms possess a remarkable capacity for bioaccumulation, allowing them to absorb nutrients and environmental contaminants from the growth substrate. These may include heavy metals such as lead, cadmium, and mercury, as well as pesticide residues [8]. For this reason, it is essential that mushroom preparations be derived from controlled cultivation systems, in which the substrate is carefully monitored to prevent contamination and ensure a consistent bioactive profile [42].

Another important safety concern is the potential misidentification of fungal species. Several toxic, even lethal, mushroom species may exhibit morphological characteristics very similar to those of edible or medicinal species, making visual identification unreliable and potentially dangerous. Accidental ingestion of toxic mushrooms mistakenly identified as edible species can lead to severe poisoning syndromes ranging from gastrointestinal toxicity to irreversible organ damage, including liver or kidney failure [43].

For these reasons, high-quality mushroom preparations should meet several essential criteria. Accurate botanical and molecular identification of the species is fundamental to guarantee the authenticity of the raw material. Modern genomic techniques are effective tools for preventing adulteration or accidental substitution with toxic species [44,45]. In addition, mushroom biomass powders should ideally be obtained through freeze-drying or low-temperature drying, processes that help preserve thermolabile compounds and maintain biological activity [46]. Finally, the use of standardized extracts enables greater reproducibility of biological effects, as the concentration of specific bioactive compounds can be controlled and kept consistent across production batches [41].

Moreover, contamination with mycotoxins produced by other fungal species may represent a potential safety concern, particularly when cultivation, storage, or processing conditions are not adequately controlled [47].

In addition to heavy metals, mushrooms are also known for their ability to accumulate radionuclides, a phenomenon widely documented following nuclear accidents such as the Chernobyl and Fukushima disasters [48,49,50]. This capacity has been partly attributed to the presence of melanin and other compounds that may enable certain fungal species to interact with ionizing radiation [51].

However, it is important to note that the mushroom species most commonly investigated in the present review, including *Ganoderma lucidum*, *Hericium erinaceus*, *Cordyceps* spp., and *Lentinula edodes*, are typically cultivated under controlled conditions [52,53,54]. This significantly reduces the risk of environmental contamination and ensures greater safety and standardization of the final products.

### 3.3. Methodological Challenges in Mushroom Research

Despite the long history of medicinal mushroom use and the growing number of publications in this field, several methodological challenges still limit the translation of experimental findings into standardized clinical applications.

One of the main difficulties lies in the heterogeneity of mushroom preparations used in experimental and clinical studies. In many cases, studies do not clearly specify which part of the mushroom has been used—mycelium, fruiting body, or whole biomass—and they also do not provide detailed information on extraction methods [32]. Since the chemical composition of mushrooms can vary significantly by species, strain, growth substrate, and processing conditions, comparing and reproducing results across studies remains challenging [55].

Another important issue concerns the intrinsic complexity of the fungal phytocomplex. While classical pharmacology often focuses on isolating single active molecules, the biological activity of medicinal mushrooms is believed to result from the synergistic interactions among multiple compounds, including polysaccharides, terpenoids, and polyphenols. This complexity has led to growing interest in network pharmacology, an approach that aims to understand how multiple bioactive molecules can simultaneously interact with diverse biological targets and signaling pathways [14,15,55].

Finally, although numerous studies have described the mechanisms of action of mushroom-derived compounds in vitro, there remains a need for well-designed randomized controlled clinical trials in humans [36]. Future research should therefore focus on the standardization of mushroom preparations and on the use of genomic and analytical techniques to enable precise identification of fungal species and the reproducibility of experimental results [45].

These methodological considerations are particularly relevant when evaluating the potential prebiotic effects of mushrooms, since the interaction between fungal polysaccharides and the gut microbiota may vary considerably depending on the structural composition and processing of the mushroom preparation [21].

## 4. Mushrooms as Emerging Prebiotics

### 4.1. What Defines a Prebiotic

The concept of prebiotics was first introduced by Gibson and Roberfroid in 1995, who defined prebiotics as “non-digestible food ingredients that beneficially affect the host by selectively stimulating the growth and/or activity of one or a limited number of bacteria in the colon” [56]. This definition highlighted two fundamental aspects: resistance to digestion in the upper gastrointestinal tract and the ability to selectively promote beneficial members of the gut microbiota.

As research on host–microbiota interactions has expanded, the definition of prebiotics has been refined. In 2017, the International Scientific Association for Probiotics and Prebiotics (ISAPP) proposed an updated definition, describing a prebiotic as “a substrate that is selectively utilized by host microorganisms conferring a health benefit” [57]. This broader definition emphasizes not only selective microbial utilization but also the resulting physiological benefits for the host, including improvements in metabolic regulation, immune modulation, and intestinal barrier function.

From a mechanistic perspective, compounds considered prebiotics typically share several key characteristics. First, they must resist digestion and absorption in the upper gastrointestinal tract so they reach the colon intact. Second, they must be fermentable by members of the gut microbiota. Finally, their fermentation should result in measurable benefits for the host, either through changes in microbial composition or through the production of bioactive metabolites, particularly SCFAs such as acetate, propionate, and butyrate [56,57].

Traditionally recognized prebiotic compounds include non-digestible carbohydrates such as inulin, fructo-oligosaccharides, and galacto-oligosaccharides. However, growing evidence suggests that a wider range of dietary substrates may exert prebiotic-like effects. In this context, edible mushrooms have attracted increasing attention as potential sources of novel prebiotic compounds [58]. Their cell walls contain complex polysaccharides—including β-glucans, chitin, and other structural fibers—that resist human digestive enzymes and can serve as fermentable substrates for gut microorganisms. These characteristics have led to the hypothesis that mushrooms may represent a promising source of compounds with prebiotic-like properties, although most of the currently available evidence derives from in vitro and animal studies, with more limited data from human clinical investigations [58,59].

### 4.2. Mushroom Polysaccharides as Fermentable Substrates

A key feature supporting the prebiotic potential of edible and medicinal mushrooms is the presence of structurally complex polysaccharides that resist digestion in the upper gastrointestinal tract [60]. In particular, mushroom cell walls contain substantial amounts of β-glucans, chitin, and other dietary fibers, which are not efficiently hydrolyzed by human digestive enzymes [9,61]. As a result, these compounds can reach the colon largely intact, where they become available for interaction with the gut microbiota.

Among mushroom-derived polysaccharides, β-glucans are the most extensively studied, owing to their abundance and recognized biological activity. However, the prebiotic properties of mushrooms cannot be attributed to β-glucans alone. The fungal cell wall represents a structurally heterogeneous matrix that also includes chitin and other non-digestible polysaccharides, which together contribute to the fermentable substrate pool available to intestinal microorganisms. This biochemical complexity distinguishes mushrooms from more conventional prebiotic ingredients based on isolated carbohydrates and supports the view of the whole mushroom as a potential source of novel prebiotic fibers [24,55,62].

Because these polysaccharides resist digestion and absorption in the upper gastrointestinal tract, they can be selectively utilized by members of the colonic microbiota. Their fermentation provides metabolic substrates for beneficial microorganisms and may favor a more diverse and metabolically active microbial ecosystem [63]. In this respect, mushroom polysaccharides serve not only as structural components of fungal biomass but also as biologically relevant dietary substrates that modulate host–microbiota interactions.

The structural diversity of mushroom fibers may be particularly relevant from a functional perspective. Different glycosidic linkages, degrees of branching, and physicochemical properties can influence microbial accessibility and fermentation patterns, thereby affecting both the composition of the gut microbiota and the generation of bioactive metabolites [39,64,65]. For this reason, mushroom polysaccharides are increasingly considered promising candidates for developing microbiota-targeted nutritional strategies [55,65,66,67].

These structural characteristics are directly linked to the microbiota-modulating effects discussed in the following section. In particular, the fermentability of mushroom polysaccharides, especially β-glucans, can influence the selective growth of beneficial microbial taxa and the production of SCFAs, as described in Section 4.3.

### 4.3. Evidence from Microbiota Studies

A growing body of experimental evidence supports the ability of edible and medicinal mushrooms to modulate the composition and metabolic activity of the gut microbiota. Most of the available data derive from in vitro fermentation models and animal studies, while human clinical trials remain relatively limited. Nevertheless, the existing literature consistently suggests that mushroom-derived polysaccharides, particularly β-glucans, can influence microbial communities in ways generally associated with beneficial health outcomes [10].

In vitro fermentation studies have shown that mushroom polysaccharides can serve as fermentable substrates for intestinal microorganisms, leading to selective changes in microbial populations [68]. In particular, several studies have reported an increase in bacterial genera commonly associated with gut health, including *Bifidobacterium* and *Lactobacillus*, which play important roles in maintaining intestinal barrier function, producing beneficial metabolites, and inhibiting the growth of pathogenic microorganisms [37].

Evidence from animal models further supports these observations. Dietary supplementation with mushroom extracts or isolated polysaccharides has been shown to modify gut microbial composition and increase the relative abundance of beneficial taxa [37]. In addition to *Bifidobacterium* and *Lactobacillus*, some studies have reported increases in *Akkermansia*, a mucin-degrading bacterium associated with improved metabolic health and enhanced intestinal barrier integrity. At the same time, mushroom supplementation has been linked to shifts in the *Firmicutes*-to-*Bacteroidetes* ratio, a microbial parameter frequently associated with metabolic and inflammatory conditions [69,70].

Despite these promising findings, clinical evidence in humans remains comparatively scarce [71]. In addition, the response of the gut microbiota to dietary interventions can vary considerably among individuals because of baseline microbiota composition, host genetics, dietary habits, and environmental factors. Moreover, the available studies show considerable heterogeneity in terms of mushroom species investigated, extraction methods, polysaccharide composition, dosage, and duration of supplementation, which may partly explain the variability observed across experimental outcomes. Differences in analytical approaches used to characterize gut microbiota composition may also contribute to inconsistencies among studies.

The limited number of human studies suggests that mushroom-derived polysaccharides may influence microbial diversity and metabolic activity, although further well-designed randomized controlled trials are needed to confirm these effects and to define the optimal species, dosage, and preparation methods [72].

Overall, available evidence, mainly derived from in vitro and animal studies, suggests that mushrooms might exert prebiotic-like effects by selectively supporting beneficial microbial populations and modulating gut microbial ecology [68]. However, the underlying mechanisms and their translation into clinically relevant dietary interventions remain to be fully elucidated [71].

### 4.4. Short-Chain Fatty Acid Production and Metabolic Effects

One of the most relevant consequences of the fermentation of mushroom-derived polysaccharides by the gut microbiota is the production of SCFAs. These metabolites are generated through the microbial fermentation of non-digestible carbohydrates that reach the colon and represent a key mechanism through which dietary fibers exert systemic physiological effects [73].

The primary SCFAs produced during microbial fermentation are acetate, propionate, and butyrate, which differ in their metabolic pathways and biological functions. Acetate is the most abundant SCFA in the colon and circulation and can serve as a substrate for peripheral tissues involved in lipid and cholesterol metabolism. Propionate is largely taken up by the liver, where it contributes to gluconeogenesis and may influence lipid metabolism and appetite regulation [74]. Butyrate, in contrast, in a in vivo study, is the preferred energy source for colonocytes and plays a crucial role in maintaining intestinal epithelial integrity [69].

Through these mechanisms, SCFAs exert several beneficial effects on intestinal physiology and systemic metabolism. In particular, butyrate helps maintain intestinal barrier integrity by supporting epithelial cell metabolism and promoting tight junction protein expression [21]. This effect helps preserve the functional barrier of the intestinal mucosa and reduces the translocation of pro-inflammatory molecules into the systemic circulation [75].

SCFAs also play an important role in immune regulation. By interacting with immune cells and modulating inflammatory signaling pathways, these metabolites can help regulate low-grade systemic inflammation and support immune homeostasis [75]. In addition, SCFAs influence several aspects of energy metabolism, including glucose regulation, lipid metabolism, and appetite control, highlighting their relevance in metabolic health [76].

Taken together, the fermentation of mushroom-derived polysaccharides and the subsequent production of SCFAs represent a key link between dietary fungal fibers, gut microbiota activity, and host physiological responses [73]. Through these mechanisms, edible and medicinal mushrooms may contribute to intestinal homeostasis and broader metabolic regulation [24].

## 5. Major Bioactive Compounds

Edible and medicinal mushrooms can be considered natural reservoirs of structurally diverse bioactive compounds due to their remarkable capacity to synthesize polysaccharides, phenolic compounds, terpenoids, and other secondary metabolites with significant biological activities. These compounds contribute to many of the physiological effects attributed to mushrooms, including immunomodulatory, antioxidant, metabolic, and microbiota-modulating activities. Understanding the chemical diversity of these molecules is therefore essential for interpreting the health-promoting properties of edible and medicinal mushrooms [21].

Medicinal mushrooms are a particularly rich source of bioactive molecules that can directly interact with the gut microbiota [3]. This interaction is not limited to local effects but also results in remodeling of the intestinal environment, enhanced immune defenses, and the production of systemic metabolites that are fundamental to host health [19]. The main molecular classes responsible for these effects are discussed in the following sections.

### 5.1. β-Glucans

β-glucans are complex structural polysaccharides composed of glucose monomers linked by β-1,3 glycosidic bonds in the linear backbone and β-1,6 bonds in the branching chains [77]. Since the human organism lacks the β-glycosidase enzymes required to cleave these linkages, these compounds resist gastric hydrolysis and reach the intestinal tract largely intact, where they exert, as demonstrated in an in vitro study, both prebiotic and immunomodulatory effects [78]. Their biological activity varies according to their solubility and structural complexity, as shown by in vitro data [79].

**Insoluble β-glucans.** Characterized by high molecular weight and high degree of polymerization, insoluble β-glucans primarily act as true novel prebiotics [78,80]. They represent a selective substrate for beneficial bacterial populations, particularly *Bifidobacterium* species, which possess specialized enzymatic systems capable of internalizing and fermenting these polysaccharides, as investigated in an in vitro study [81]. This metabolic process results in the substantial production of SCFAs, including acetate, propionate, and butyrate, as evidenced by both animal models and in vitro studies [80,82]. These SCFAs exert crucial physiological roles: they provide energy for colonocytes, promote mucosal regeneration, strengthen epithelial tight junctions, and markedly reduce the translocation of pro-inflammatory endotoxins into the systemic circulation [82].

**Soluble β-glucans.** Soluble fractions are believed to be absorbed in the ileum through pinocytosis mediated by M cells and subsequently transported to the mucosa-associated lymphoid tissue (MALT). By binding to specific membrane receptors such as Dectin-1 and CR3 on macrophages, neutrophils, and natural killer cells, these polysaccharides may act as Biological Response Modifiers (BRMs), influencing innate immune responses [61,83]. Experimental studies suggest that β-glucans can activate mechanisms associated with trained immunity (TRIM), inducing epigenetic and metabolic reprogramming that enhances the responsiveness of innate immune cells to subsequent stimuli [84]. However, most of the current evidence derives from in vitro experiments and animal models, and the extent to which these mechanisms occur in humans following dietary mushroom consumption remains under investigation.

### 5.2. Polyphenols

Medicinal mushrooms contain significant concentrations of polyphenolic compounds, particularly flavonoids, which are synthesized as part of the fungal defense system against oxidative stress and parasitic organisms. Approximately 90–95% of high-molecular-weight polyphenols are not absorbed in the small intestine and therefore reach the colon largely intact [17].

At this stage, a bidirectional interaction with the gut microbiota occurs: polyphenols can modulate and selectively influence the composition of the intestinal microbial community [85], while gut bacteria metabolize these complex molecules, breaking them down into smaller, low-molecular-weight compounds [17,78].

These microbiota-derived metabolites can subsequently be absorbed into the systemic circulation, where they exert pronounced antioxidant and anti-inflammatory cellular activities that are considered important for protection against chronic degenerative diseases [86].

### 5.3. Trehalose and Chitin

The gut microbiota can also be influenced by other structural compounds present in mushrooms, particularly trehalose and chitin [87].

**Trehalose**. Often referred to as the “mushroom sugar”, trehalose is a disaccharide used by fungal cells to stabilize proteins and preserve cellular viability under extreme environmental conditions [88]. At the intestinal level, it can act as a well-tolerated prebiotic substrate, while its systemic effects may be even more relevant. Trehalose has been reported to activate lipolysis and induce cellular autophagy, thereby reducing insulin resistance and limiting the accumulation of visceral and hepatic fat [16]. Moreover, trehalose can cross the blood–brain barrier (BBB) and has been associated with potential neuroprotective effects [89]. Due to its resistance to hydrolytic degradation, it does not generate advanced glycation end products (AGEs), suggesting a favorable safety profile even in conditions of altered glucose metabolism [90].

**Chitin**. Chitin is a polymer of N-acetylglucosamine that constitutes the rigid structural framework of the fungal cell wall [91]. As an insoluble dietary fiber in the digestive tract, chitin can increase fecal bulk, improve intestinal peristalsis, and contribute to mechanical detoxification. Through its interaction with the intestinal mucosa, chitin may also help reduce the absorption of excess sugars, lipids, and potentially harmful substances [62].

### 5.4. Species-Specific Compounds

The adaptogenic efficacy of mycotherapy is further enhanced by secondary metabolites characteristic of specific mushroom species that interact with various physiological systems beyond the intestinal environment.

**Cordycepin** (from *Cordyceps*). Cordycepin is a nucleoside analogue of adenosine that acts as a potent post-transcriptional epigenetic modulator [92]. By substituting for adenosine, it interferes with the polyadenylation of inducible mRNA transcripts, thereby inhibiting the synthesis of proteins involved in inflammatory processes and reducing the production of pro-inflammatory cytokines such as IL-1β, IL-6, and TNF-α [93]. In addition, cordycepin has been reported to exert significant metabolic effects, including inhibiting adipogenesis, promoting white adipose tissue browning, and activating the AMPK signaling pathway [94]. Through these mechanisms, it has been investigated as a potential therapeutic target for metabolic disorders, including hepatic steatosis and insulin resistance [87,95].

## 6. The Gut–Brain Axis and the Role of Mushroom-Derived Metabolites

The interaction between medicinal mushrooms and the human organism extends well beyond the gastrointestinal tract, potentially influencing neurological and psychological functions [96,97]. Current scientific evidence allows these systemic effects to be interpreted within the framework of a complex functional architecture in which the gut microbiota and its metabolites act as biochemical messengers linking the intestine and the brain [98].

### 6.1. The Gut–Brain Axis

The GBA is a bidirectional communication network that connects the central nervous system with the enteric nervous system [19]. This continuous dialogue occurs through three main pathways: neural signaling (primarily via the vagus nerve), endocrine communication (through the hypothalamic–pituitary–adrenal axis), and immune mechanisms mediated by cytokine modulation [99].

Within this framework, the gut microbiota acts as a primary modulator of brain function [100]. A condition of eubiosis supports intestinal barrier integrity and helps reduce systemic inflammatory burden. Conversely, dysbiosis can alter intestinal permeability, allowing endotoxins and pro-inflammatory molecules to enter the bloodstream [101,102]. These circulating mediators may reach the central nervous system and contribute to neuroinflammatory processes, potentially leading to dysregulation of the physiological stress response and negatively affecting mood, cognitive functions, and even circadian rhythms [103,104].

### 6.2. Microbiota-Derived Metabolites

The modulation of GBA by medicinal mushrooms is closely linked to the ability of their dietary fibers, particularly complex polysaccharides, to be fermented by intestinal bacteria, thereby producing bioactive metabolites [24].

Among the most relevant metabolites are SCFAs. In addition to nourishing intestinal epithelial cells and strengthening tight junctions to prevent endotoxemia, SCFAs exert neuroactive effects: they can cross the BBB and influence neurogenesis, BBB integrity, and neurotransmitter synthesis [105].

Furthermore, the maintenance of specific bacterial populations, such as *Bifidobacterium* and *Lactobacillus*, promoted by the prebiotic activity of mushroom-derived compounds, is crucial for regulating inhibitory neurotransmitters like GABA [106]. Adequate GABA levels in the frontal and parietal cortex are considered important for protecting against cognitive decline and neurodegenerative disorders such as Alzheimer’s disease [107].

### 6.3. Mushrooms and Modulation of the GBA

Several medicinal mushrooms have been described as functional “brain-supporting foods”, acting on the GBA to modulate neuroinflammation, promote neuroplasticity, and support cognitive and behavioral functions [3]. The main biological effects of selected medicinal mushrooms, together with their major bioactive compounds are summarized in Table 1.

***Hericium erinaceus***. This species is widely considered one of the most relevant mushrooms for nervous system support [108]. At the intestinal level, it helps reduce inflammation and restore mucosal integrity [109]. At the systemic level, its active metabolites (hericenones and erinacines) can cross the BBB and stimulate nerve growth factor synthesis [110]. This process has been associated with enhanced neurogenesis and with the mitigation of anxiety- and depression-related states [111].

***Lentinula edodes*** (Shiitake). By remodeling the microbiota, this mushroom may exert protective effects on the GBA. In murine models (in vivo), Shiitake-derived compounds, including β-glucans and vesicle-like nanoparticles, have been shown to improve cognitive function and prevent neuroinflammation, partly by modulating intestinal permeability, endotoxemia, and tryptophan metabolism [112,113]. These effects are associated with the restoration of brain-derived neurotrophic factor (BDNF) levels and improved synaptic structure in key brain regions such as the hippocampus and prefrontal cortex [112].

***Ganoderma lucidum*** (Reishi). This species appears to influence the GBA by modulating neurotransmitter balance and controlling intestinal inflammation [114,115]. Its adaptogenic properties have been associated with improvements in sleep quality and with the regulation of hypothalamic function, contributing to the attenuation of systemic neuroinflammatory states [116,117].

***Cordyceps militaris***. This Emerging evidence suggests that *Cordyceps militaris* may influence the GBA through both intestinal and systemic mechanisms. At the gut level, it has been shown to improve intestinal barrier integrity in pigs, modulate the microbiota composition, and increase SCFA production, thereby reducing local inflammation [118]. In animal models, these effects have been associated with improvements in cognitive performance, as well as reductions in anxiety- and depression-like behaviors under stress conditions [119,120,121]. Although direct clinical evidence remains limited, these findings support a potential role of *C. militaris* as a modulator of the microbiota–GBA.

**Table 1:** Major bioactive compounds in selected medicinal mushrooms and their main biological effects.

Mushroom Species	Major Bioactive Compounds	Main Biological Effects	References ^[a]^
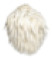 *Hericium erinaceus*	Erinacines, hericenones	Neuroprotection; stimulation of nerve growth factor (NGF); modulation of GBA	[108,109,110,111]
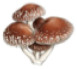 *Lentinula edodes*	β-glucans (lentinan), polysaccharides	Immunomodulation; prebiotic effects; enhancement of SCFA production	[112,113,122]
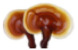 *Ganoderma lucidum*	β-glucans, triterpenoids	Immunomodulation; anti-inflammatory activity; gut microbiota modulation	[114,115,116,117]
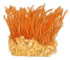 *Cordyceps militaris*	Cordycepin, polysaccharides	Metabolic regulation; anti-inflammatory effects; modulation of gut microbiota	[118,119,120,121]

^[a]^ References are provided for the main biological effects listed in the table. For further biological activities, see the individual studies and review articles cited in the main text.

## 7. Discussion

The growing body of literature on edible and medicinal mushrooms suggests that these organisms may represent a promising class of functional foods capable of modulating host physiology through multiple biological mechanisms. Unlike conventional pharmacological approaches that primarily target established disease conditions, mushroom-derived compounds are increasingly investigated for their potential role in disease prevention and health maintenance.

In particular, the present review highlights the relevance of mushroom polysaccharides as fermentable substrates capable of modulating gut microbial composition and metabolic activity [24,70,71].

Through their interaction with the intestinal microbiota, these compounds may stimulate the production of SCFAs, metabolites known to support intestinal barrier integrity, modulate immune function, and regulate metabolism. These mechanisms support the hypothesis that edible mushrooms may act as emerging prebiotic foods, influencing host physiology through microbiota-mediated pathways.

In addition to modulating the microbiota, several mushroom-derived metabolites have been reported to interact with immune, metabolic, and neurological signaling pathways. Compounds such as β-glucans, polyphenols, trehalose, and species-specific metabolites, including cordycepin and erinacines, may influence inflammatory processes, oxidative stress responses, and cellular metabolic regulation [9,36,39].

These effects are increasingly interpreted through the framework of network pharmacology, which considers the synergistic activity of multiple bioactive compounds acting simultaneously on diverse biological targets.

Particularly relevant is the potential role of mushrooms in modulating the GBA [19,99]. Through microbiota-derived metabolites and direct neuroactive compounds, some mushroom species have been associated with mechanisms that may influence neuroinflammation, neuroplasticity, and neurotransmitter regulation. However, most available evidence comes from in vitro experiments and animal models, and the number of well-designed human clinical trials remains limited.

Another important aspect concerns the quality and standardization of mushroom preparations. As highlighted in this review, significant variability exists in the composition of commercial mushroom products depending on the fungal species, the anatomical part used (mycelium or fruiting body), cultivation conditions, and extraction techniques. Such variability may influence both the concentration of bioactive compounds and the reproducibility of biological effects. For this reason, rigorous characterization of mushroom preparations and standardized production methods are essential for translating experimental findings into clinical applications.

Another limitation concerns the heterogeneity of mushroom preparations used across studies, including differences in fungal species and experimental models.

Future research should therefore focus on well-designed randomized clinical trials to evaluate the effects of specific mushroom species, standardized extracts, and defined dosages on gut microbiota composition and host metabolic outcomes. Advances in metagenomics, metabolomics, and systems biology may further help clarify the complex interactions among mushroom-derived compounds, microbial metabolism, and host physiology.

## 8. Conclusions

Edible and medicinal mushrooms represent a promising and still underexplored source of natural prebiotic compounds. Their complex matrix of polysaccharides, polyphenols, and other bioactive metabolites may modulate the composition and metabolic activity of the gut microbiota, particularly by stimulating SCFA production.

Through microbiota-mediated mechanisms and specific secondary metabolites, mushrooms may influence several physiological systems, including immune regulation, metabolic homeostasis, and the GBA. These properties support the growing interest in mushrooms as functional foods that can contribute to health maintenance and disease prevention.

Despite the growing number of experimental studies, further research is required to clarify the clinical relevance of these effects. Future investigations should prioritize standardized mushroom preparations, clearly defined experimental protocols, and well-controlled human clinical trials. Such studies will be essential for establishing evidence-based recommendations regarding the use of edible and medicinal mushrooms in nutrition and preventive medicine.

## Data Availability

No new data were created or analyzed in this study. Data sharing is not applicable to this article.

## Abbreviations

The following abbreviations are used in this manuscript:

TCM	Traditional Chinese Medicine
SCFAs	Short-Chain Fatty Acids
GBA	Gut–Brain Axis
BBB	Blood–Brain Barrier
